# On the Therapeutic Action of Bismuth

**Published:** 1861-06

**Authors:** J. B. M’Caw

**Affiliations:** Professor of Chemistry and Pharmacy


### SELECTIONS.

*On the Therapeutic Action of Bismuth.* An Extract from a Lecture Delivered at the Medical College of Virginia, by J. B. M'Caw, M. D., Professor of Chemistry and Pharmacy.

Having concluded my remarks on bismuth and its combinations, I call your attention for a few moments to their value in the treatment of disease. I think you will see how much the curative virtues of these compounds depend upon the physical and chemical properties, already explained to you.

First, then, remember the physical properties of the bismuth compounds and especially of the basic subnitrate, the usual form in which this agent is administered by the physician. This salt, as I have told you, is the result of the action of water on the ternitrate, causing a white, almost insoluble semi-unctuous precipitate, which is the subnitrate of the pharmacopœia.

Known as the magistery of bismuth, this powder was much prized by the ladies, long ago, as a cosmetic, because of its emollient influence on the skin, concealing pimples, freckles and other deformities to which even the fair sex is sometimes liable, and leaving a beautiful white gloss much to be admired. Not only did its external application act soothingly to the excoriated or irritated integument, but it was also used as a drying powder, checking a too profuse perspiration, by coating over, with its delicate varnish, the exuding membrane.

Suppose then, you have a patient with dyspepsia, an irritable mucous membrane, painfully excited during the act of digestion, by the excessive flow of the gastric juice and the mechanical efforts of the muscular coat, can you not, with great propriety, appeal to the innocuous and emollient subnitrate of bismuth, which coats over the angry membrane with its soft white precipitate, shields it from all causes of irritation, and gives to the digestive function a great amount of comfort?

Recollecting, also, its mechanical effect upon the transpiration of the skin, you can see why the subnitrate should be made use of in the treatment of serous diarrhœas, especially those not the result of organic disease; in the "summer disease" of teething children, and in the irritable form of disordered bowels following measles and scarlet fever. Here, as we have to act upon a great extent of surface, it is necessary to give the remedy in large repeated doses, and in such cases I have prescribed one ounce in twenty-four hours with great advantage.

So much for the mechanical influences of this agent on the mucous membrane, explaining, to some extent, its therapeutic action; but I think by observing its chemical attributes you may also derive some hints of practical importance.

I have shown you that the presence of bismuth or any of its compounds can be detected by the agency of sulphuretted hydrogen, when a precipitate of the brown-black sulphide of bismuth will at once appear. So sensitive is the subnitrate to the presence of this agent that its use as a cosmetic has almost been abandoned since the introduction of the coal gas as an illuminating agent, which, when impure, often sets sulphuretted hydrogen free. The pearly cheeks of a cluster of beauties decked for the ball would soon, under the action of this unerring detector, even rival the sooty complexion of Christy's Minstrels.

If you have to treat hereafter cases of gastric or enteric disorder accompanied by disengagements of sulphuretted hydrogen, as, for instance, that distressing condition of stomach called "egg belch," or the windy colic of defective digestion and torpid liver, remember that subnitrate of bismuth may be relied on to neutralize the offending and offensive gas by the formation of a sulphide which is entirely innocuous, if not positively curative. The dark color of the stools after the administration of this remedy proves the truth of this statement, as there is always enough sulphuretted hydrogen to give the characteristic precipitate.

The surgeon as well as the physician may gain some useful ideas from a study of the physical and chemical properties of bismuth. As an application to burns, or diseases of the skin, such as erysipelas, the subnitrate, with its soothing influence, will be found very valuable; also in excoriations of the skin, and the chafing of infants, the result of acrid discharges. As a disinfectant, I would advise you to use this harmless salt freely, being equally effective and greatly more convenient than the tar and plaster of Velpeau, or the permanganates of Gridwood—a fœtid ulcer, a sinus connected with a carious bone, or a malignant sore, pouring out its putrid odors, will be greatly improved under the free application of the agent under discussion.

Before closing these remarks, which are not intended to infringe upon the prerogatives of our colleagues of surgery and the practice, but rather to show you the value of chemistry even in the more practical departments of medicine, I will mention that as arsenic is not unfrequently found in combination with bismuth in a native state it would be well before using the subnitrate, in the large quantities I have recommended, to make sure that your preparation is pure. The usual mode of manufacturing the article, I should think, would preclude the possibility of much danger from this source, but we have the high authority of Prof. R. E. Rogers, of Philadelphia, to justify us in giving you this caution. In experiments made by him some years ago he found traces of arsenic in many of the samples of subnitrate of bismuth collected by him from the druggists of Philadelphia. As far as my own observations go, however, I have never detected arsenic in the subnitrate in any appreciable quantity, and never heard, after an experience of many years of its producing the symptoms of arsenical poisoning in a single instance. —*Maryland & Virginia Medical Journal.*

